# Environmental and Genetic Traffic in the Journey from Sperm to Offspring

**DOI:** 10.3390/biom13121759

**Published:** 2023-12-07

**Authors:** Pallav Sengupta, Sulagna Dutta, Fong Fong Liew, Vidhu Dhawan, Biprojit Das, Filomena Mottola, Petr Slama, Lucia Rocco, Shubhadeep Roychoudhury

**Affiliations:** 1 Department of Biomedical Sciences, College of Medicine, Gulf Medical University, Ajman 4184, United Arab Emirates; 2 School of Life Sciences, Manipal Academy of Higher Education (MAHE), Dubai 345050, United Arab Emirates; 3 Department of Preclinical Sciences, Faculty of Dentistry, MAHSA University, Jenjarom 42610, Selangor, Malaysia; 4 Department of Anatomy, All India Institute of Medical Sciences (AIIMS), New Delhi 110029, India; 5 Department of Life Science and Bioinformatics, Assam University, Silchar 788011, India; 6 Department of Environmental, Biological and Pharmaceutical Sciences and Technologies, University of Campania Luigi Vanvitelli, 81100 Caserta, Italy; 7 Laboratory of Animal Immunology and Biotechnology, Department of Animal Morphology, Physiology and Genetics, Faculty of AgriSciences, Mendel University in Brno, 613 00 Brno, Czech Republic

**Keywords:** genetic infertility, epigenetic changes, transgenerational effects, semen quality, environmental pollution

## Abstract

Recent advancements in the understanding of how sperm develop into offspring have shown complex interactions between environmental influences and genetic factors. The past decade, marked by a research surge, has not only highlighted the profound impact of paternal contributions on fertility and reproductive outcomes but also revolutionized our comprehension by unveiling how parental factors sculpt traits in successive generations through mechanisms that extend beyond traditional inheritance patterns. Studies have shown that offspring are more susceptible to environmental factors, especially during critical phases of growth. While these factors are broadly detrimental to health, their effects are especially acute during these periods. Moving beyond the immutable nature of the genome, the epigenetic profile of cells emerges as a dynamic architecture. This flexibility renders it susceptible to environmental disruptions. The primary objective of this review is to shed light on the diverse processes through which environmental agents affect male reproductive capacity. Additionally, it explores the consequences of paternal environmental interactions, demonstrating how interactions can reverberate in the offspring. It encompasses direct genetic changes as well as a broad spectrum of epigenetic adaptations. By consolidating current empirically supported research, it offers an exhaustive perspective on the interwoven trajectories of the environment, genetics, and epigenetics in the elaborate transition from sperm to offspring.

## 1. Introduction

Environmental and lifestyle variables can have a profound impact on the phenotypes of future generations [1]. Male fertility is also heavily affected by environmental factors [2]. Although environmental exposures can have a detrimental influence on human health at any time, there are sensitive developmental windows, such as the prenatal, early childhood, and puberty periods, during which every possible consequence is amplified [3]. Since the beginning of evolutionary theory, it has been accepted that environmental factors determine the phenotype at the population level. A paradigm change was brought about by the discovery that parental influence may have an impact on the offspring of later generations through mechanisms unrelated to hereditary factors [4,5]. For a very long time, women have been thought to be primarily responsible for the health of their offspring, considering the paternal preconception environment to be inconsequential. Although intergenerational inheritance is a mechanistically complex process that would require epigenetic information to be maintained throughout the disruptive process of epigenetic reprogramming during gametogenesis, carried in gametes, and delivered to embryos at fertilization and then influence embryonic development, asserting intergenerational inheritance in mammals has proven to be challenging. Furthermore, the so-called “Weismann barrier”, which limits the passage of information from somatic cells to germ cells and hence to the embryo, has caused many to question the inheritance of acquired traits [6]. Due to the extreme size disparity between sperm and oocytes, paternal contributions to intergenerational epigenetic transmission have been elusive. However, recent research indicates that paternal exposure to environmental factors also influences fetal development and the health of the offspring [1].

Evidence suggests that epigenetics, together with the genetic conformation of spermatozoa, determines how an embryo will develop [7]. A growing body of research indicates that preconception exposure to specific environmental and lifestyle factors, such as diet, alcohol consumption, physical activity levels, and smoking, among others, can change the epigenetic blueprint of spermatozoa in a way that affects the phenotype of succeeding generations [7,8,9]. A study on paternal effects with only the male partner being exposed to a specific environment before conception suggested that sperm-borne factors responsive to lifestyle changes can modulate the developmental process of the offspring through epigenetic inheritance, the direct modification of the gametic epigenome by the environment, and subsequent transmission to the next generation [7].

The cellular epigenetic landscape has a higher level of plasticity than the genome, making it more susceptible to environmental influences. Paternal epigenetic alterations are thought to be caused by three main mechanisms: miRNA expression, histone modification, and DNA methylation [10]. It is critical that future studies emphasize the important influence of environmental variables on paternal germ cells throughout both the lifetime of an individual and those of future generations [11,12]. For the etiology of human diseases, understanding the process of intergenerational inheritance is crucial [13,14]. Numerous prevalent metabolic disorders, including obesity, insulin resistance, and diabetes, are influenced by a patient’s environment and lifestyle in addition to their genetics [15,16,17]. Genetic variation can only account for a small portion of the heritability of these conditions; it is now more widely recognized that epigenetic inheritance most certainly plays a vital role in these disorders [18].

There is substantial scope for further research to explore the possible links between the health outcomes of the offspring and paternal factors like age, exposure to environmental factors, and genetic and epigenetic configurations. It is only in recent times that the mechanism of inheritance began to be comprehended. At the present time of high demand for assisted reproduction, in certain cases, even spermatozoa from testicular biopsies are utilized to fertilize oocytes [19]. A comprehensive understanding of the factors that initiate molecular modifications in spermatozoa during their development and post-testicular maturation in the epididymis is essential. It is crucial to examine their implications for fertilization, embryonic development, and subsequent phenotypic manifestations in progeny. This review endeavors to elucidate the mechanisms through which environmental determinants influence male reproductive potential and concisely highlight the evidence suggesting that paternal exposure to environmental elements can be transmitted to offspring, in view of both genetic and epigenetic alterations.

## 2. Paternal Influence on Offspring Development: The Underlying Mechanisms

During the past decade, there has been a surge in studies that have shown the paternal impact on fertilization, early embryonic development, and offspring health [20,21]. The increase in paternal exposure to various pharmaceutical drugs, toxins, xenobiotics, radiations, pesticides, and dietary as well as other lifestyle factors has impacted vulnerable sperm beyond affecting the sperm quality and fertility potential to jeopardizing fetal development and offspring health [22,23]. While there is substantial evidence in the literature explaining the paternal impact on offspring health, the evolution of these paternal effects is being brought to the surface.

The paternal effect refers to a biological phenomenon in which the genotype or phenotype of the father exerts an influence on the phenotype of the offspring without altering its genotype. This effect can manifest in two primary forms: an adaptive manifestation, potentially conferring a survival advantage to the offspring, or a non-adaptive manifestation, which may have detrimental consequences or represent a neutral by-product of underlying biological processes [24].

### 2.1. Non-Adaptive Paternal Effects

The paternal influence may not invariably confer advantages; it could manifest as a secondary outcome of other processes or even have harmful effects. For instance, fathers of an advanced age may transmit epigenetic modifications through spermatozoa that negatively impact the progeny’s developmental processes. Such alterations could be associated with pathological conditions or senescence in the paternal figure, and they may not necessarily provide any adaptive benefit to the offspring [25]. The mechanisms behind this transgenerational inheritance have remained enigmatic. A recent review elaborated on advancing age in men leading to decreased fertility, lower testosterone, and reproductive pathologies, alongside increased sperm DNA damage and genetic mutations in offspring, contributing to diseases like Apert syndrome and schizophrenia [25]. These effects are linked to mutant stem cell expansion and oxidative stress (OS) impacting sperm and hormonal cells. Antioxidants could be a therapeutic strategy, pending clinical trial validation [25].

The initial skepticism about the paternal influence on offspring stemmed from the lack of clear mechanisms explaining how environmental influences could modify the genomic DNA that is passed on to the offspring [26,27]. The exposure of the sperm to environmental insults, psychological stressors, and adverse events predisposes the sperm genome to impending oxidative attack, increasing the activity of repetitive elements and thus inducing nuclear and mitochondrial DNA damage, the accumulation of mutagenic base adducts, changes in gene expression, and epigenetic changes that are transmitted to the oocyte at fertilization [28,29]. These non-adaptive factors are detrimental to the health of the offspring. They are protected in the sperm genome by various chromatin modifications, the methylation of sperm DNA, and a repertoire of sperm non-coding RNAs (sRNA, piRNA, miRNA, etc.) [27,30,31]. The paternal lineage is thus responsible for transmitting more than just its DNA. Epigenetic marks are delivered by sperm to the zygote, with evidence pointing toward the involvement of DNA methylation, histone modifications, and non-coding RNAs (ncRNAs) [32].

### 2.2. Paternal Effects as Beneficial Adaptive Responses for Offspring

Parental effects on the phenotype of the offspring can be influenced by the effect of the environment or phenotype of both the mother and father [33]. This implies that the paternal effect might have evolved as a beneficial adaptation, allowing fathers to pass on certain environmental or conditional information to their offspring without changing their genetic code [33]. It can allow the offspring to be better suited to their environment or the current circumstances. For example, if a father has experienced a particular environmental condition, this might affect the sperm in a way that primes the offspring for similar conditions, giving them a potential survival advantage [33]. This “phenotypic plasticity”, also described as “transgenerational plasticity”, is due to the effects of the parental environment and not the offspring environment on the phenotype of offspring [33].

The impacts of paternal exposure on the offspring phenotype have been the subject of much speculation, but it is not clear whether these effects are “adaptive”. One explanation for this was given as the “thrifty phenotype” hypothesis [34], where it was stated that a compromised in utero environment might program the offspring for a similar environment after birth and increase their predisposition to metabolic disorders. The transmission of paternal environmental effects increases susceptibility to diseases after birth [35].

The transmission of information on the environmental effects faced by the parents may provide an adaptive advantage to the offspring [36]. Such adaptive effects can be termed “anticipatory parental effects (APEs)”, where the parents modify the phenotype of the offspring with environmental changes to increase the fitness of both the parents and offspring [37]. However, the adaptive paternal effects may not only be APEs: the response to environmental stimuli may also decrease offspring fitness in order to achieve long-term fitness benefits by the parents, described as “selfish parental effects”, in order to achieve long-term fitness [38]. Another type of adaptive effect is described as “bet-hedging parental effects”, which occur in parents who may randomly adapt to a varying environment by creating phenotypic diversity in the offspring [38]. This evolutionary learning of the adaptation to varying environments has been described as “positive transgenerational feedback”, where the parental phenotype is progressively reinforced in successive generations [39]. The above two effects thus highlight that parental effects may not always increase offspring fitness [37]. The variation seen in the offspring phenotype exerts a greater influence on the population structure than the variation in offspring number [40].

The ability of females to affect the phenotype of the offspring by adaptive maternal effects is well documented, but the contribution of non-genetic adaptive paternal effects has been brought to the surface [27,41]. The literature has highlighted the role of transgenerational epigenetic effects in the male germ line that are transmitted via the male germ line to the offspring. Males can adjust the sperm phenotype in response to local conditions, but the transgenerational consequences of this plasticity are unknown. Increasing speculation arose on how males adjust the sperm phenotype in response to the environment, and the existence of “adaptive” paternal effects was proposed as the “thrifty telomere hypothesis” [42].

### 2.3. Paternal Effects to Mediate Sexual Conflict

The inheritance of paternal and maternal genomes creates a conflict between males and females over allele expression at heterozygous loci in the offspring [43]. Genomic imprinting, an epigenetic phenomenon, determines the expression of an allele according to its parental origin [44]. The difference in the methylation status of gametes generates an inherent asymmetry in the maternal and paternal genomes that drives differential parent-of-origin gene expression. This violates Mendel’s rules at the level of expression. Imprinting is thus a maladaptive phenomenon, as there is a loss of diploidy and the presence of uniparental disomy, and a heterozygote for one defective allele may pose a problem if there is silencing of the active allele.

The evolution of genomic imprinting has been explained by three theories [45]: (a) kinship theory [46,47], (b) sexual antagonism theory [48], and (c) maternal–offspring co-adaptation theory [49]. The theories proposed above address different fundamentals but rest on one shared feature, which is the presence of asymmetry or conflict in the maternal and paternal alleles over gene expression at heterozygous loci in the offspring [45].

Genomic or sexual conflict is not the only mechanism of imprinting: various molecular mechanisms have also been described based on the fact that the maternal and paternal alleles have distinct epigenetic marks [50].

### 2.4. Paternal Effects to Control Selfish Genetic Elements

Selfish genetic elements, or SGEs, have been referred to by various names, including selfish genes, ultra-selfish genes, selfish DNA, parasitic DNA, and genomic outlaws [51]. These are specific segments within a genome that can promote their own survival over other genetic material. They do this by biasing their own transmission, ensuring that they are passed down through successive generations at a higher rate compared to other segments of the genome [51]. A notable aspect of SGEs is that they can create a sort of conflict within the genetic material. This happens because while the SGEs are working to increase their own representation, they might be acting against the interests of the genome as a whole. This conflict may or may not have a negative impact on an individual’s health or ability to survive and reproduce. Interestingly, this phenomenon of intragenomic conflict, where parts of the genome are in competition with each other, is also a characteristic of something known as sexually antagonistic (SA) alleles [52]. These are specific gene variations that might benefit one sex while being detrimental to the other, leading to a similar type of tension within the genome. Both selfish genetic elements and sexually antagonistic alleles exemplify the complex interactions and competitions that can occur within the genetic material of an individual [52].

Replicative mobile elements, or transposable elements (TEs), are the most common SGEs and include DNA sequences that have the potential to move to new locations in the host genome [52,53]. Other groups of SGEs include segregation distorters (meiotic drivers), which target gametogenesis by killing/modifying maternally inherited endosymbionts (which either kill or feminize females, e.g., mitochondria) [52]. Genomic conflicts thus arise, as not all genes are inherited in the same way.

The prime targets of SGEs are gametogenesis and reproduction to facilitate enhanced transmission. They may increase the mutation rate and affect the evolution of genes, genomes, gene expression, sex chromosome formation, and turnover and have also been seen to affect sexual behavior [52,54,55]. Since SGEs are ubiquitous, they also affect sexual selection, including mate preferences and conflict [55]. SGE carriers are thus seen to frequently have reduced gamete production [56]. Gametogenesis is especially affected in men who have different types of SGEs and have been seen to show reduced sperm production [57].

Another well-cited example of SGEs is “selfish mitochondria”, where the conflict is between uniparentally (usually but not always maternally) inherited mitochondria and other biparentally inherited nuclear genes. Uniparental inheritance reduces the ability of selfish mitochondria to spread and is usually maternal, as the mutation rate is lower in female gametes than in male gametes [58,59].

## 3. Environmental Influences: Relating Paternal Fertility Factors and Offspring Health

### 3.1. Environmental Factors and Male Fertility: State-of-the-Art Knowledge

For the last 70 years, scientists have been studying whether fathers’ exposure to certain agents can affect their children’s health. Recently, there have been a lot of studies about how chemicals in our environment and our lifestyle habits can have an impact on our health for several generations [60,61]. Scientists have learned more about how this happens at a molecular level. This means that although we have seen the harmful effects of industrial chemicals on different generations of animals in laboratories, we do not have enough proof yet to know for sure that the same is happening in humans [62].

Environmental pollution is a major cause of male infertility in today’s world. This is due to the universal presence of environmental toxins. The quality of semen is a predictor of the outcome of male fertility [63]. Semen quality is unfavorably affected by environmental pollutants, which impairs the normal process of spermatogenesis, steroidogenesis, and sperm function, therefore decreasing the quality of fertility in males [64,65]. Anthropogenic factors such as industrial wastes, insecticides, pesticides, herbicides, etc., have adverse effects on the natural spermatogenesis process in adult males. The exposure of a male to air pollutants, certain chemicals, heavy metals, high heat, and OS results in fertility issues in the male.

Air pollution is a major cause of male fertility problems, with its sources ranging from anthropogenic (vehicle exhaust, factories, man-made fire, oil refineries) to natural activities (volcanic eruptions). Air pollutants resulting in human fertility problems include particulate matter, nitrogen oxides, ozone, carbon monoxide, sulfur dioxide, etc. In the initial phases of spermatogenesis, gaseous pollutants like sulfur dioxide and nitrogen dioxide have a dramatic negative impact on sperm motility and concentration that is found to be more aggressive [66]. Automobile exhaust pollutants, like nitrogen oxide and lead, significantly decrease sperm motility, forward progression, and sperm kinetics [67]. Exposure to ozone leads to a decrease in the overall sperm count in males [68]. Particulate matter (PM_2.5_) results in an increase in the number of sperm cells with cytoplasmic droplets and morphological abnormalities in sperm heads [69].

Exposure to several endocrine-disrupting chemicals (EDCs), such as heavy metals, pesticides, dioxins, bisphenol, phthalates, etc., also affects male fertility [70]. Heavy metals like lead, cadmium, arsenic, barium, mercury, etc., affect semen quality in men by adversely affecting sperm viability and morphology [71,72]. Heavy metals generate reactive oxygen species (ROS), which cause lipid peroxidation and DNA damage in sperm, thereby resulting in infertility problems among men [73,74]. Lead- and cadmium-containing compounds alter hormone levels, thus causing the impairment of semen quality [75]. Exposure to high levels of copper sulfate and cadmium chloride significantly reduces sperm motility [76].

Bisphenol A (BPA) is a ubiquitous environmental toxicant and is widely used in the production of polycarbonates, epoxy resins, dental sealants, compounds, etc. Studies conducted to explore the effects of BPA on male reproductive health have shown inconsistent results but have shown adverse effects on semen quality, hormonal profile, fecundity, and fertility [77]. BPA has estrogenic, anti-androgenic, and antithyroid activities and, hence, disrupts the HPG axis [78]. Increased exposure to BPA results in sperm DNA damage, decreased sperm motility and count, and an increased risk of aneuploidies in sperm [78,79].

Pesticides, especially dibromo chloropropane and ethylene dibromide, are known to cause direct spermatozoa damage, the alteration of Leydig cell function, the disordered endocrine function of hormonal regulation during the synthesis, release, storage, transport, and clearance of hormones, the binding of hormones to their receptors, the function of the thyroid gland, etc., thereby leading to male infertility [80].

Phthalates, commonly found in cosmetics, some medicines, toys, etc., cause a wide range of male reproductive organ dysfunctions known as “phthalate syndrome”, consisting of diminished anogenital distance, low sperm count, infertility, undescended testes, hypospadias, and other reproductive tract anomalies [81]. Exposure to phthalates can cause a reduction in semen volume, total sperm count, sperm concentration, and morphological abnormalities in the sperm head.

Exposure to excessive heat can also lead to infertility issues in males. An adequate temperature is crucial in maintaining normal spermatogenesis in the testes. The scrotal temperature is 2–4 °C lower than the normal core body temperature [82,83]. Any factor that increases the scrotal temperature will negatively affect the process of spermatogenesis [84]. It is also observed that a mere rise of 1–1.5 °C in scrotal temperature can result in the impairment of spermatogenesis, leading to various semen abnormalities, such as oligozoospermia, azoospermia, or teratozoospermia [85].

Studies also link cell-phone use with male infertility [86,87]). Kim et al. reported that exposure to cell phones is associated with a reduced sperm volume and concentration [87]. Kesari et al. concluded that electromagnetic radiation exposure can damage Leydig cell function and can lead to a reduction in the testosterone level, seminiferous tubule shrinkage, and a decrease in sperm count as well as motility [86].

OS might be the reason for decreased fertility and can be caused by contact with different environmental substances that lead to the excessive generation of ROS. While some ROS are necessary for optimal sperm function, excessive amounts can damage sperm via lipid peroxidation, DNA, and chromatin integrity, leading to fertilization failure and pregnancy loss [88]. Spermatozoa themselves produce limited ROS, as they lose most of their cytoplasm during development; however, external factors such as toxins can increase seminal ROS levels [89]. Lifestyle factors like smoking, obesity, and stress contribute to OS, adversely affecting male fertility [8,90,91]. Commonly used products can also hinder sperm motility by causing mitochondrial ROS and DNA damage [92]. Environmental genotoxins, including titanium dioxide nanoparticles, have been shown to fragment sperm DNA via ROS [93]. Animal studies support OS as a primary cause of sperm DNA damage; for instance, formaldehyde in rats [94] and arsenic in mice lead to genotoxicity [95]. Additionally, toxic substances like sulfur mustard cause DNA alkylation and ROS production [96]. Damaged sperm DNA can reduce sperm count due to apoptosis and is linked to recurrent pregnancy loss because of genetic defects affecting the embryo [89,97,98].

### 3.2. Paternal Exposure to Environmental Factors: Transmission to the Offspring

Environmental factors not only directly affect individuals but also have intergenerational consequences, with parents potentially transmitting these effects to their offspring (Table 1). Maternal malnutrition during pregnancy can impair the physical and cognitive development of the offspring [99], while maternal smoking raises the risks of preterm birth, low birth weight, and infant mortality [100]. The implications of paternal environmental exposures on progeny are not fully elucidated, yet there is growing evidence that such exposures may negatively influence offspring health and development. Research indicates that paternal contact with environmental toxins, like heavy metals, bisphenols, dioxins, pesticides, and air pollution, is linked to adverse health outcomes in children, including behavioral and physical disorders [101,102]. Lead or mercury exposure in fathers is associated with a heightened risk of autism and attention-deficit hyperactivity disorder (ADHD) in their children [103], as well as obesity and type 2 diabetes [104]. Paternal drug use and stress are connected to cognitive and behavioral changes in children [105,106]. Additionally, nicotine exposure in fathers is known to cause behavioral alterations across generations [107]. Similarly, paternal exposure to cannabis [108] and certain pesticides [109,110] increases the risk of cognitive and behavioral issues in offspring.

Moreover, paternal exposure to air pollution and smoking has been linked to cardiovascular and respiratory diseases, cognitive deficits, and abnormal fetal development in descendants [115,116], and fathers’ occupational contact with toxins may lead to cancer in their children [120].

The underlying mechanisms of how paternal exposures affect offspring health are complex and involve epigenetic modifications—changes in gene expression that do not alter the DNA sequence but can be triggered by environmental conditions [121]. Epigenetic alterations in sperm DNA resulting from paternal exposure to toxins are passed on to offspring, influencing their gene expression and development. Behavioral changes in children may arise from disturbances in DNA methylation, altering sperm RNA profiles and potentially causing depression and anxiety phenotypes [121]. Paternal cigarette smoke exposure has also been observed to increase global sperm DNA methylation, disrupting metabolic functioning in offspring [122].

## 4. Genetic Causes of Spermatogenesis Disturbances

### 4.1. Microdeletions of Azoospermic Factor AZF Region

Couple infertility ranges between 12.6 and 17.5% [123], out of which half of the cases are attributed to the male factor due to low sperm concentration or poor spermatozoa quality [124]. Among the major causes of male infertility are genetic causes, like chromosomal aberrations and microdeletions of the Y chromosome. These genetic causes result in azoospermia and severe oligospermia in males [125].

The Y chromosome plays a crucial role in the human genome because it contains the SRY gene (Sex-determining Region Y), which is crucial for the development of male characteristics [126]. The presence of the SRY gene triggers the pathway that leads to the development of testes, which, in turn, produce male hormones and initiate the development of male reproductive structures. It consists of two pseudoautosomal regions (PARs), the short (Yp) and long (Yq) arms [126]. In the Yq 11.23 interval, the human spermatogenesis locus is present [127]. Upon the molecular characterization and genetic dissection of Yq11.23, three non-overlapping, distinct subregions were identified. These subregions are coined as azoospermic factors (AZFs) a, b, and c, each of which carries genes involved in spermatogenesis and the maturation of sperm [128].

The AZFa subregion is situated within the proximal region of the deletion interval 5 of Yq11.21 (5C subinterval of Y chromosome) [129,130]. The region encompasses approximately 800 kilobases and encodes single-copy genes exhibiting homology with the X chromosome [128]. These genes are essential for the process of normal spermatogenesis. The AZFa locus includes a set of candidate genes, namely, ubiquitin-specific peptidase 9, Y-linked (USP9Y), dead box on Y (DBY), and ubiquitously transcribed tetratricopeptide repeat gene, Y-linked (UTY) [131].

The AZFb subregion is situated in the central region of Yq11, between the 5M and 6B subintervals [130]. This subregion spans from 6.2 to 7.7 Mb and overlaps with the AZFc subregion by 1.5Mb [132]. This region has many different types of genes, including some having only a single copy and others having multiple copies. The AZFb region contains genes that code for proteins, which include lysine (K)-specific demethylase 5D (KDM5D), eukaryotic translation initiation factor 1A, Y-linked (EIF1AY), ribosomal protein S4 Y isoform 2 (RPS4Y2), and chromosome Y open reading frame 15A and 15B (CYORF15) [133,134].

The AZFc genetic locus is positioned at the distal region of the Yq chromosomal arm, with a deletion span comprising subintervals 6C through 6E [130]. The AZFc domain extends 4.5 Mb and is situated within three extensive palindromic sequences that originate from six distinct amplicon groups [135]. The AZFc locus is responsible for encoding a total of 21 candidate genes and 11 families of transcription units that exhibit exclusive expression in the testis [135]. Within the AZFc deletion interval, a total of seven families can be identified, which include genes such as Golgi autoantigen, golgin subfamily a2-like, Y-linked 1 (GOLGA2LY1), and chondroitin sulfate proteoglycan 4-like and Y-linked pseudogene 1 (CSPG4LYP1) [135]. Significant candidate genes within this deletion interval comprise DAZ and BPY2, as well as CDY1 (specifically CDY1a and CDY1b on Y chromosome 1) [134,136]. The AZF microdeletion is linked not solely to azoospermia but rather to a diverse range of testis histological profiles, spanning from Sertoli cell only (SCO) to hypo-spermatogenesis [135]. The investigation of testicular histology has revealed that the deletion of AZFa is correlated with a complete lack of germ cells and the existence of Sertoli cells within the seminiferous tubules. This manifestation is indicative of the SCO syndrome. The elimination of AZFb has been linked to the cessation of germ cell development at the pachytene stage [137]. Conversely, AZFc deletion has been associated with the interruption of germ cell development at the spermatid stage and has also been observed to result in hypo-spermatogenesis and maturation arrest with consequent azoospermia or oligozoospermia [138]. For more than twenty years, the STS-PCR method has been utilized and greatly developed to become the top-rated laboratory examination procedure for detecting microdeletions in the Y chromosome [133,134].

### 4.2. Paternal Genetic Diseases and Defects

#### 4.2.1. Klinefelter Syndrome

Klinefelter syndrome (KS) (47, XXY) affects one in every 660 men and is the most prevalent chromosomal anomaly in men (>4%). It was described for the first time in 1942 [139,140]. KS was discovered to be caused by a supernumerary X chromosome in a man in 1959. Alterations in the nuclear structure that induce infertility in KS may be caused by the presence of two alleles of several genes associated with the X chromosome, which usually function according to the theory of disomy and do not undergo inactivation following extra-chromosomal lyonization. An abnormal karyotype has been seen in 10% of adults with azoospermia and 5–6% of adults with oligozoospermia as well [89,141].

KS is not directly inherited and may arise at random in the egg or sperm. According to Jo et al., KS affects about 10% of azoospermic males, and morbidity occurs at a rate of 0.1–0.2% in the general population, while the syndrome is detected in 0.15–0.17% of prenatal diagnoses. During the prenatal stage, almost 18,000 pregnant women were tested for KS in their offspring, and it was reported that the risk of KS in children rises as the mother becomes older. If the mother is 35 years old, the chances that she will give birth to a son with KS are somewhat higher. Trisomies are induced by meiotic nondisjunction, which can arise when combined X chromosomes do not separate in paternal and maternal meiotic nondisjunction. This chromosome has additional genes that prevent testicular growth and result in less testosterone [142,143].

KS exhibits a broad spectrum of phenotypes, and it is not defined by any solitary symptom. This complexity may partly explain why there are various disorders being evaluated in conjunction with KS. Furthermore, the patient’s age plays a critical role in determining the emergence of specific signs and symptoms. With the escalation in the number of characteristics and comorbidities that accumulate over time, as well as the deterioration of existing ones, the phenotype typically becomes more severe as the individual ages [144]. The projected incidence of KS is anticipated to reflect this complex interplay of factors, although additional research may be necessary to fully understand the trends and implications [144].

The emergence of the clinical characteristics of KS enables grouping according to the underlying pathogenetic mechanism (Table 2) [144,145]. Most of the boys and men with KS are unaffected, and they can have normal, healthy lives. With KS, the lower testosterone levels pose several issues. Infertility is the main issue; however, there are ways to treat it. An adequately titrated testosterone dosage in those patients should be given special consideration since they often have a minor testosterone shortfall, especially those with a moderate phenotype [146]. Long-term effects, however, may include muscle and bone mineral mass loss, an elevated risk of type 2 diabetes, and the threat of metabolic syndrome. The loss of germ cells in KS begins during pregnancy, persists through childhood, and becomes more severe throughout puberty. Long-term germ cell degeneration is associated with seminiferous tubule fibrosis and a decrease in testis size [147].

Low blood testosterone levels are linked to a poor metabolic profile, implying a unique unifying mechanism for previously separate discoveries that low testosterone levels and decreased mitochondrial activity induce insulin resistance in males [148]. It implies that KS is associated with increased insulin resistance and high rates of type 2 diabetes (T2DM) [149]. More KS individuals exhibited increased fasting plasma insulin levels, and insulin sensitivity calculations have shown a substantial reduction in insulin sensitivity [150]. Davis and co-workers have also found that 30% of KS patients have cardiometabolic risk factors with significantly elevated fasting triglycerides and low HDL cholesterol. The body mass index (BMI), blood pressure, and fasting glucose levels of the groups, on the other hand, did not differ [151].

In individuals with KS, the beginning of T2DM marks a turning point in the progression of their cardiovascular risk profile. Subclinical systolic, diastolic, and vascular dysfunctions, which, in turn, contribute to cardiopulmonary impairment and increased morbidity and death in these individuals, may be affected. KS individuals also tend to be more susceptible to cardiovascular diseases [150,152];. Jørgensen et al. revealed that KS treated with TRT had shorter QTc intervals (QTc) compared to controls, but untreated and hypogonadal KS had intervals equivalent to controls [153]. Patients with greater levels of differentially expressed genes (DEGs) had substantially lower QTc periods. This effect was considerably stronger in men who inherited the extra X chromosome from their fathers. Furthermore, testosterone levels in the blood did not correlate with QTc times [154]. Fricke et al. discovered a prevalence of mitral valve prolapse (MVP) of 55% [155]. Pasquali et al. discovered a significant increase in isovolumic relaxation time and mitral deceleration time, as well as a decrease in the E/A ratio and pulmonary vein velocities, consistent with mild diastolic dysfunction, with no differences between treated and untreated KS patients. Notably, males on testosterone therapy who had secondary hypogonadism did not have normal cardiovascular parameters [156].

The frequency of bone diseases, especially lower bone mineral density, is likewise rising in KS patients. This phenomenon is a result of higher bone turnover and an elevated risk of bone fractures. In contrast to fractures in elderly men, the effects of KS on the physical and socioeconomic elements of men are more active and more dangerous. When normal aging occurs, the mechanism of reduced bone mineral density changes, since in KS patients, hypogonadism occurs during important pubertal phases in the formation of bones, accompanied by poor physical strength and muscular strength [157].

The association between psychiatric disorders and KS patients was addressed by assessing the attitudes of the participants toward problems such as the sense of stigma, the unfavorable effects of the karyotype XXY, and children. Almost 70% of male patients with KS showed signs of sadness, including anxiety and schizophrenia, psychoses, hallucinations, and paranoid illusions. They concluded that there was an elevated risk of mental problems for both adolescents and adults with this condition [158].

Men with KS are not at an increased risk of having malignant neoplasia. Breast cancer, lung cancer, non-Hodgkin lymphoma, and extragonadal germ cell tumors mostly situated in the mediastinum, on the other hand, are more common. A number of meta-analyses on the prevalence of male breast cancer have revealed that KS is the most important independent breast cancer risk factor in males, accounting for 4 to 30 times the risk in normal men [159,160,161,162,163]. By contrast, they are significantly less likely to develop prostate cancer [163,164]. Autoimmune rheumatic disorders are generally recognized to be more frequent in women due to increased estrogens compared to androgens. Because of the additional X chromosome, which causes fewer androgens and estrogens in individuals with KS, the frequency and autoimmune risk in men with the syndrome are enhanced [165].

#### 4.2.2. Kallmann Syndrome

Kallman syndrome is a rare genetic disorder that affects approximately 1 in every 8000 males [166]. Kallman syndrome causes males and females to experience delayed puberty and sexual immaturity, as well as other physical abnormalities [167]. Dysfunction of the HPG axis leads to the interruption of the normal spermatogenesis process, which is due to the reduced or absent secretion of GnRH. Patients with this syndrome may also suffer from a reduced ability to smell (hyposmia) or a complete loss of their sense of smell (anosmia) because of olfactory bulbs that are either missing or not fully formed [166].

Kallman syndrome is also known as congenital hypogonadotropic hypogonadism (CHH) with anosmia/hyposmia [168]. In 1856, Mastre De San Juan, a Spanish pathologist, made a significant observation linking hypogonadism and anosmia. He made this discovery while examining a male cadaver with a micropenis, underdeveloped testes, and no pubic hair that lacked olfactory bulbs [169]. In 1944, Franz Josef Kallman, a geneticist from the USA, published research indicating that genetic factors are responsible for underdeveloped sexual traits and the loss of sense of smell. He described it as a condition that presents numerous irregularities. The condition that was subsequently identified was named Kallman syndrome [170].

The GnRH released by the hypothalamus in humans stimulates the synthesis of pituitary gonadotrophins (LH and FSH). LH and FSH then act as gonadotropins to synthesize gonadal hormones and spermatogenesis in the testes. LH stimulates the Leydig cells of the testes to produce testosterone, and FSH stimulates the Sertoli cells of the testes, which requires testosterone for receptor induction to control the process of spermatogenesis; GnRH deficiency results in deficient LH and FSH synthesis, thereby disturbing the normal process of testicular function and spermatogenesis [171]. Both olfactory neurons and GnRH-secreting neurons originate from the brain development area known as the olfactory placode. Both types of neurons pass through the olfactory bulb to migrate to the hypothalamus. The olfactory bulb is responsible for the origin of smelling sensations. Dysgenesis of the olfactory bulb results in a disturbance in GnRH neurons’ progression toward the hypothalamus, thereby directly resulting in anosmia/hyposmia and abnormal LH and FSH due to a lack of GnRH secretion. This abnormal LH and FSH secretion also result in delayed puberty and underdeveloped secondary sexual characteristics in both males and females [172,173,174].

Genetic mutations are the underlying cause of CHH. It can occur intermittently or run in families. The pattern of heredity within family cases may follow autosomal dominant, autosomal recessive, or X-linked modes [175]. To date, researchers have linked two different genes, *KAL1* on chromosome Xp22.3 and *FGFR1* on chromosome 8p11.23, to this condition [122]. While X-linked KS (XKS) is caused by *KAL1* mutations and autosomal dominant KS (AKS) can be attributed to *FGFR1* mutations, these two genes only account for around 20–24% of all cases of KS [176]. Anosmin1, a glycoprotein that is secreted in diverse extracellular matrices, is specified by KAL1, while FGFR1, a member of the receptor tyrosine kinase superfamily that binds fibroblast growth factor 2 (FGF2) and several other FGF ligands, is specified by FGFR1. During the development of the olfactory–GnRH system, two proteins called anosmin1 and FGFR1 are present and have a role in regulating neuronal migration and the elongation and branching of their axons [177,178,179]. Genetic mutations affecting these specific genes can result in the malformation of the olfactory tract, which may also cause developmental issues in other tissues observed in individuals with KS, such as cleft lip, cleft palate, dental agenesis, synkinesia, and renal agenesia [180,181,182,183,184]. Other genes such as *NSMF*, *FGFR1*, *FGF8*, *FGF17*, *IL17RD*, *PROK2*, *PROKR2*, *HS6ST1*, *CHD7*, *WDR11*, *SEMA3A*, *TUBB3*, and *SOX10* are linked to KS-encoded proteins, and they work together with anosmin 1—a protein associated with the migration of GnRH neurons [185,186,187].

## 5. Epigenetic Markers Transmitted to Offspring

Epigenetic transmission is sometimes relegated mainly to the paternal contribution, i.e., it occurs through the germ cells and through several generations [188]. These heritable epimutations, such as DNA methylation/acetylation, histone modifications, and small RNAs contributing to heritable phenotypic variation, play a very important role in evolution [26]. However, these variations can also generate disease states by introducing spontaneous mutations through the germline over generations [10]. The most worrying issue concerning generational toxicological processes is that the effects of toxic exposures are visible after several generations. Exposure to various pesticides has led to serious effects, such as testicular and prostate diseases, tumors, and motor impairments, only in the F2 and F3 generations [189], raising concerns for future unexposed generations.

DNA methylation is one of the main epigenetic markers, and any perturbation in this process will cause effects on subsequent generations in the same way that the alteration of the sperm RNA expression profile can affect offspring health [190].

### 5.1. DNA Methylation and Acetylation of Sperm

DNA methylation is often linked to gene silencing, and a connection has been shown between histone deacetylation and DNA methylation. However, the overall effect on the position and length of methylation zones in the promoter and in the gene-coding region is still relatively uncertain [7]. The transfer of non-genetic factors by sperm in response to environmental challenges may contribute to alterations and affect epigenetic systems in the paternal germline. Various approaches or manipulations used in paternal effects include the modulation of the stress exposure time frame. Stress typically has significant physical consequences that might have a negative impact on the germline and male fertility [7,27]. Potential genome defense strategies against such mutagenic agents include DNA methylation, chromatin modifications, and the production of short RNAs (sRNAs). At the translational and post-translational stages, all three components are known to be involved in modulating the potential impacts as potential carriers of epigenetic inheritance [7,27].

The biological process is determined by interactions of a methyl or acetyl group with the DNA molecule that can change the genomic transcriptional activity without affecting its genetic coding. The degree to which sperm is transmitted from father to son varies considerably from the mother to predominantly paternal specimens [27]. Both histone acetylation and DNA methylation in spermatogenesis are well investigated for their role in transferring non-genetic information between generations. In combination with histone changes, DNA methylation has a crucial role in regulating gene expression within germ cells, contributing to three key processes: (i) specifying and forming primordial germ cells, (ii) eradicating and restoring germ-specific patterns in embryos and sexual patterns during gametogenesis, and (iii) establishing genomic patterns during gametogenesis [7,191].

Almost as soon as DNA was introduced as a genetic material, methylation was discovered in animals. Whilst many researchers hypothesized that DNA methylation affects gene expression, numerous studies in the 1980s showed that gene regulation and cell differentiation are involved in DNA methylation. DNA methylation is now universally accepted as a key epigenetic process, together with other regulators, that affects gene activation [192].

DNA methylation, the transfer of a methyl group from S-adenyl methionine (SAM) to the fifth carbon of a cytosine residue, mediated by DNA methyltransferases, modulates gene expression, silences transposons and endogenous retroviral sequences, inactivates the X chromosome, and affects genomic imprinting [193]. In eukaryotes, gene expression may be governed in a variety of ways, but the methylation of DNA is a typical epigenetic signaling mechanism that cells utilize to keep genes in the “off” state. The methylation of cytosine at CpG sites has been related to reduced fertility and disease promotion in offspring. Environmental exposures have been related to a range of diseases later in life, and changes in sperm DNA methylation have been found as biomarkers for these exposures. Although higher histone retention in sperm following protamine replacement and non-coding RNAs have been related to male infertility, DNA methylation is the most important epigenetic biomarker [193].

Lujan et al. discovered a male infertility signature of DNA methylation areas in male infertility patients. Male idiopathic infertility patients were shown to have a hallmark of differential DNA methylation regions (DMRs). This new application of epigenetic biomarkers to distinguish responsive from non-responsive patient groups will improve clinical treatment for male infertility patients [194].

Non-CpG methylation increases inside and around B1 SINE transposon sequences in male germ cells during mouse fetal development, indicating that sperm cells have both CpG and non-CpG methylation. Non-CpG methylation has been seen in paternally methylated areas as well as several CpG islands, where methylation is highest at birth. Such dynamic methylation change patterns (de novo methylation followed by methylation loss as spermatozoa mature) contrast sharply with CpG methylation dynamics. During epigenetic reprogramming, the partial removal of DNA methylation marks creates a biological temporal window in which environmental influences can be passed down from generation to generation. Several genomic characteristics, such as L1HS transposons, are resistant to epigenetic reprogramming because they are heavily methylated throughout germline development [195,196].

In retrotransposon sequences, however, reprogrammable genomic areas appear to be missing. While the functional consequences of methylation at the proximal ends of protein-coding regions are apparent, the significance of methylation at transposons or repeated repetitions is undefined and difficult to anticipate by nature. The functional study of genes near escapees in human primordial germ cells indicated an enrichment for genes expressed in the brain and influencing neural development [197].

Differential DNA methylation in sperm around genes important in neurogenesis regulation and central nervous system development has been reported in several studies. External stimuli cause epigenetic alterations. It is worth noting cross-generation research that focused on the metabolic readings of descendants related to tolerance to glucose and changes in gene expression in metabolism. The nature and specificity of the (metabolic) phenotypic reading could not be accurately determined. It is probable that the diverse environmental exposures utilized may differently influence other phenotypic reactions, for example, behavioral features. In intergenerational research, the expansion of the panel of the phenotypic characterization of children with a parallel profile of the epigenetic blueprint in spermatozoa would help to establish a linkage between particular sperm epigenetic modifications and implications for offspring [7,27].

### 5.2. Histone Retention and Modifications

Histones are basic proteins that attach to DNA in the nucleus and allow it to condense into chromatin [193,198]. Histone modifications can enhance or decrease the binding of regulatory factors to DNA, resulting in decreased or increased gene activity and expression. Histone methylation and acetylation, for example, are known to have a function in mammalian spermatogenesis and development [193,198]. The length of the protruding tails of histone proteins influences how tightly the DNA is wrapped. Histones control gene expression through post-translational modifications like acetylation and methylation. Histone hyperacetylation is involved in histone removal, and acyl-CoA bioavailability is thought to influence sperm genome compaction [191,199]. Butyrylation, another post-translational histone modification, can occur concurrently with histone hyperacetylation during spermatogenesis, decreasing acetylation-dependent histone removal and delaying replacement by protamines, resulting in chromatin compaction control. Thus, environmental variables may influence chromosomal conformation in mature spermatozoa by influencing acyl-CoA availability, acetylation, and butyrylation [198,200,201,202].

In almost every species studied, from plants to humans, the environmentally mediated epigenetic transgenerational transmission of disease and phenotypic variation has been established [203]. This non-genetic form of inheritance is passed down to subsequent generations via epigenetic alterations in the sperm and/or egg [203]. The mechanisms controlling differentially methylated areas, non-coding RNA (ncRNA), and differential histone retention co-regulation are uncertain. In control lineage generations, Skinner et al. revealed a highly conserved collection of histone retention sites that did not change much between generations [203].

Protamines replace 90% (in humans) to 95% (in house mice) of histones during spermatogenesis, and the remaining histones may undergo post-translational modifications that affect gene expression at these loci [204,205]. Except for paternally derived histone retention regions, where protamines are removed and replaced by maternally derived histone, highly compacted sperm chromatin is repaired afresh after conception. Paternally retained histones have the potential to interact with imprinted genes, affecting the epigenetics and transcriptomes of future embryonic cells. The paternal histone retention changes in sperm provide a pathway for epigenetic inheritance, which has been found in offspring sperm. Environmental variations in sperm histone retention are thought to impact the epigenetic transgenerational inheritance of parent-of-origin allelic transmission of paternally derived sperm epimutations and diseases [204,205,206,207,208].

Histones are found in genic regions, distal intergenic areas, repeats, and retrotransposons. The estimation of their specific genomic distribution in sperm may vary due to differences in the endonuclease concentration or digestion time [209,210,211,212,213]. They are particularly retained at promoter sequences recognized by CCCTC-binding factor (CTCF) and near genes vital for embryonic development. After gamete fusion, maternal nucleosomes replace protamines, but residual paternal histones remain linked to the paternal genome. Histone retention is common in regions with a high CpG density and low DNA methylation, including promoters of housekeeping and development-regulating genes. Additionally, histones in sensory perception genes may reflect a biological mechanism that allows the paternal environmental damage to affect sensory perception in offspring. Genomic areas with retained histones are enriched for differentially methylated regions in sperm from obese versus thin men. These observations highlight the specific role of histone retention in embryo development and its potential sensitivity to lifestyle and environmental factors affecting sperm development [209,210,211,212,213].

The presence of an enrichment mark for H3Kme273 in repeated regions of the sperm genome suggests that histone retention is regulated by silencing repetitive sequences. Following that discovery, the concept of early gene expression in the embryo was shown. The fact that histones are maintained at random in infringement patients, as well as the fact that both H3K4me and H3K27me marks were decreased, suggests that histone positioning and modification are critical for normal sperm function [206,210,214].

Histone tails frequently contain a positive charge and so securely attach to negatively charged DNA. Acetylation reduces the charge on DNA, making it less tightly coiled and increasing transcription. During spermatogenesis, nuclear histones are mostly replaced by protamine, and histone lysine residues are acetylated before being removed from chromatin [200]. The acetylation of lysine results in a weak histone-to-negatively charged DNA interaction, resulting in increased chromatin fluidity [214]. Some infertile men have abnormalities in protamine content as well as DNA methylation in sperm, and there is also a negative association between the DNA methylation level and sperm motility or the proportion of the normal form [215,216]. Verma et al. reported that trimethylated histone 3 lysine 27 (H3K27me3) varied at multiple genomic regions involved in sperm function and embryonic development in water buffalo [217]. H3 post-translational changes (H3K27ac and H3K27me3) in the sperm head had different spatiotemporal patterns. Methylation levels were higher than acetylation levels, and the two fertility groups were inversely related. H3-interacting proteins were involved in methylation regulation, nucleosome assembly, DNA replication control, and chromatin assembly, among other subcellular functions [218].

The gradual protamination of sperm DNA during spermatogenesis passively erases the epigenetic signals transmitted by the deleted histones [219]. Protamination, like DNA demethylation during spermatogenesis, therefore, contributes to epigenetic modifications. Environmental variables that influence protamination and protamine placement may thus create an epigenetic signal in and of itself, which is equally important in regulating transcriptional activity after fertilization as histone modification and DNA methylation changes [219,220]. Matrix attachment regions (MARs), which constitute an additional layer of chromatin structural information in sperm, are connected to the nuclear matrix alongside protamine- and histone-bound DNA. MARs are essential for normal embryonic development and have been functionally related to DNA replication and the formation of the male pronucleus following fertilization [219,220,221].

### 5.3. Sperm-Borne Small RNAs

Small RNA molecules that are not converted into proteins are known as non-coding RNAs [222]. They have an essential function in controlling the expression of genes. It has been clear in the past few years that the functioning of germline cells is reliant on the presence of small RNAs. These molecules influence numerous biological processes, including spermatogenesis. The altered expression of small RNAs plays a role in male infertility, resulting in decreased sperm concentration and motility as well as altered sperm morphology [222].

Paternal inheritance via the transfer of RNA from sperm has been extensively documented [223]. Sperm cells possess a class of diminutive RNA molecules referred to as sperm borne small RNAs (sRNAs). These small RNAs have been demonstrated to be crucial in controlling the expression of genes through epigenetic mechanisms, particularly during the initial phases of embryo development [224].

Sperm cells contain a variety of RNAs, including messenger RNA (mRNA), transfer RNA (tRNA), ribosomal RNA (rRNA), and small non-coding RNAs. Of the various sRNA groups, the ones most thoroughly researched within sperm cells are the microRNAs (miRNAs) and piwi-interacting RNAs (piRNAs). miRNAs are single-stranded, small RNAs consisting of 18–24 nucleotides that regulate gene expression by binding to the 3′ untranslated regions of target mRNAs, thereby targeting mRNAs for translational inhibition or degradation. piRNAs are small RNAs that are longer than miRNAs (consisting of 26–31 nucleotides), and they regulate transposable elements in germline cells [225].

Despite having significantly less RNA, several RNAs found in sperm can trigger certain reactions. There is mounting proof that RNA plays a role in signaling and activating oocytes in the initial stages of zygote formation [226]. Additionally, there is growing support for the idea that these RNAs may contribute to the transmission of paternal epigenetic traits [227]. Sharma et al. showed that the transfer of miRNA from father to offspring can influence the gene expression involved in glucose homeostasis regulation [228]. Grandjean et al. showed that male mice exposed to a high-fat diet have offspring with changes in gene expression involved in glucose and lipid metabolism [229]. Chen et al. showed that tsRNAs present in sperm contribute to the inheritance of metabolic disorders in the offspring [230]. They also showed that piRNA expression in sperm is linked to male infertility and may contribute to transgenerational effects on reproductive health and fertility. Gapp et al. reported that sperm borne RNAs are involved in the transgenerational inheritance of the effects of early trauma on neurodevelopmental outcomes in offspring [105]. Yuan et al. identified piRNAs in human seminal plasma, suggesting a potential role in transgenerational epigenetic inheritance and the regulation of gene expression in offspring [231]. Sendler et al. showed that smoking influenced the small non-coding RNAs in human sperm, suggesting a potential role in transgenerational effects on epigenetic modifications and gene regulation in offspring [232]. Rodgers et al. revealed that psychological stress in parents leads to alterations in the contents of microRNA, which showed a transgenerational effect on the behavior of offspring [233]. Fullston et al. showed that due to diet and paternal obesity, sperm microRNAs are altered, which influences the metabolic health of the offspring. The offspring are more susceptible to metabolic disorders [234]. Benchaib et al. [235] reported that abnormal miRNAs in sperm are associated with male infertility and can be potentially transmitted to the offspring, which can influence fertility and reproductive health (Table 3).

**Figure 1:**
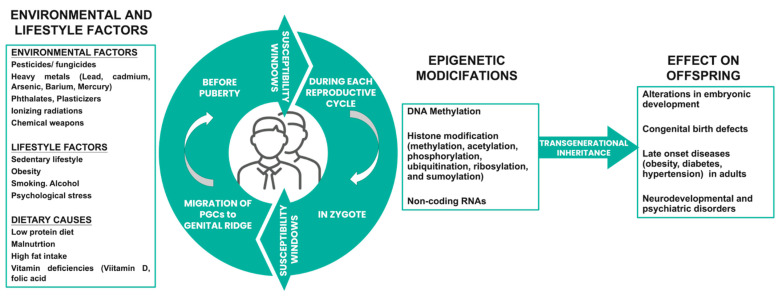
Effects of environmental factors and epigenetic modifications of the father on the offspring.

Figure 1 summarizes the effects of environmental factors and epigenetic modifications on the offspring. 

## 6. Impact of External Factors on Epigenetics of Sperm: The Connecting Link

The phrase “sperm factor” may be deceptive since it oversimplifies what is obviously a complex combination of variables. As a result, one of the questions is, “how are the different mechanisms connected?” The genotype–phenotype connection of KS is mostly unclear at the moment [236]. Only one gene, SHOX, has been related to a specific phenotypic feature of KS. The phenotypic and varied expressivity of KS cannot be explained by a single genetic etiology, according to the available KS evidence. Evidence indicates that the increase in or deletion of the X chromosome in humans causes epigenetic instability that can contribute to the phenotype exhibited in patients with aneuploid sex chromosomes by changing transcriptional amplification regulations [236,237,238].

Male infertility has a major effect on environmental factors before conception and is one of the most prevalent reproductive diseases [239]. For a long time, it was assumed that epigenetic changes do not span generational boundaries. Scientists hypothesized that the epigenetic memory gained during life is completely lost during the development of sperm and egg cells [239]. Several studies have demonstrated that epigenetic markers may be passed down through generations, but how they influence offspring is still under investigation. Another study has found that in addition to hereditary DNA, hereditary epigenetic instructions have a role in controlling gene expression in offspring. Changes in DNA or its packaging components that affect gene expression, effectively turning gene transcription on and off, are passed down to daughter cells [239].

Several environmental variables, such as pollution, stress, and nutrition, have an impact on human health, particularly through epigenetic pathways. It is generally understood that the epigenome serves as a link between the genome and the environment, and that epigenetic markers may be passed down through generations [240]. During germ cell development, epigenetic signatures begin to be established in the testes and continue to the maximum level of complexity in spermatozoa [241]. Surprisingly, such a signal does not remain constant throughout spermatozoa development along the epididymis, but rather varies with time. The epithelial epididymal cells provide a significant contribution here, which is explained by epididymosomes [242].

Current problems include establishing whether the various epigenetic components work independently or interactively and how these interactions, and the ensuing consequences may be context dependent. Future research is required to better understand the molecular mechanisms behind this. Animal models can be useful in disentangling the strands.

## 7. Conclusions and Future Directions

The current evidence-based study highlights the various environmental factors affecting fertility in males and various mechanisms and hypotheses associated with the same. Evidence from the current literature has shown that the effects of the pre-conception exposure of sperm to environmental insults are mediated by the gametic transmission of environmentally driven epigenetic information. The current array of genetic diagnostics in infertility is limited to assessing a broad spectrum of infertility, and attempts should be further made to explore novel genetic and epigenetic diagnostics, for example, seminal biomarkers. This new and emerging domain of transgenerational epigenetics has been explored by numerous researchers, and there exists an unmet need for further explicit attention to concerns around various environmental regulations and health policies.

Ongoing preconception environmental research in paternal epigenetics is focused on DNA methylation and non-coding RNAs. But the identification of other sensitive environmental epigenetic mechanisms, such as chromatin structure and 3-D conformation, histone modifications and enrichment, exosomal non-coding RNAs, and their expression in seminal plasma and epididymosomes, will hold importance in the future. The emerging discipline of three-dimensional (3-D) genomics is the most recent and promising domain which assesses the 3-D conformation and functional regulation of intranuclear genomes, such as DNA replication, DNA recombination, gene expression regulation, transcription factor regulation mechanism, and the maintenance of the 3-D conformation of genomes. This will help in the assessment of key genes and signal pathways in various diseases.

This evidence-based study comprehensively discussed how the genetic and epigenetic (DNA methylation) marks created in response to environmental stress events exert a post-fertilization function in affecting the phenotype of the offspring. The assessment of the effect of these DNA methylation patterns on the developmental programming of the embryo is very challenging. The emergence of genome-editing tools like the CRISPR-Cas9 system, for example, CRISPR-Cas9 fused to DNA methyltransferase 3A (DNAMT-3A) or demethylation-participating enzyme TET1, may further be used.

## Figures and Tables

**Table 1:** Environmental traffic from father to offspring.

Environmental Factors	Evidence of Paternal Transmission	Health Outcomes in Offspring	References
Heavy metals (mercury, lead, etc.)	Epigenetic changes in sperm DNA	Autism, attention-deficit hyperactivity disorder (ADHD)	[111,112,113,114]
High-fat diet/obesity	Changes in sperm DNA methylation	Obesity, type 2 diabetes	
Psychological stress/drugs	Changes in sperm DNA methylation and RNA expression	Anxiety, depression, behavioral abnormalities	[105,106]
Air pollution/cigarette smoking	Epigenetic changes in sperm DNA	Cardiovascular diseases, respiratory diseases, cognitive impairment, reduced growth of fetus	[115,116]
Radiation	Epigenetic changes in sperm DNA	Cancer	[117,118]
Pesticides	Epigenetic changes in sperm DNA	Birth defects, behavioral abnormalities, cognitive impairment	[110,119]

**Table 2:** Emergence of clinical characteristics of Klinefelter syndrome according to underlying pathogenetic mechanism.

	PATHOGENETIC MECHANISMS AND CLINICAL CHARACTERISTICS		
	Supernumerary X Chromosome	Testosterone Deficiency	Supernumerary X Chromosome and Testosterone Deficiency
ONSET	Before puberty	At puberty/adulthood	Before puberty, with progressive worsening after puberty
SIGNS	Longer legs Small testes Congenital malformations (cleft lip, cleft palate, hernia)—rare	Sparse body and facial hair; decreased muscle mass; female pubic escutcheon; bilateral gynecomastia; eunuchoid skeleton; longer legs (due to testosterone deficiency in fetal life); impaired estradiol/testosterone ratio	Tall stature; eunuchoid skeleton; gynecoid pelvis; elevated FSH/LH; increased BMI (overweight/obese); metabolic abnormalities; reduced bone mineral density; genital abnormalities at birth (rare)
SYMPTOMS	Disability in speech and language; azoospermia	Erectile dysfunction; reduced libido; weakness; impaired well-being	Mood disturbances

**Table 3:** Transgenerational epigenetic markers.

Epigenetic Marker(s)	Target Gene(s)/Pathway(s)	Findings	References
miRNAs	Metabolic genes	Paternal transfer of miRNAs contributed to the inheritance of diet-induced obesity and metabolic disorders in offspring.	[229]
tRNA fragments	Gene expression	tRNA fragments in sperm played a role in gene expression regulation during fertilization and embryonic development.	[228]
tsRNAs	Metabolic genes	Sperm tsRNAs contributed to the transgenerational inheritance of acquired metabolic disorders in offspring.	[230]
Sperm-borne RNAs	Neurodevelopmental genes	Sperm-borne RNAs were implicated in the transgenerational inheritance of the effects of early trauma on neurodevelopmental outcomes in offspring.	[105]
piRNAs	Epigenetic regulation	piRNAs were identified in human seminal plasma, suggesting a potential role in transgenerational epigenetic inheritance and the regulation of gene expression in offspring.	[231]
Small non-coding RNAs	Epigenetic modifications	Smoking influenced the small non-coding RNAome in human sperm, suggesting a potential role in transgenerational effects on epigenetic modifications and gene regulation in offspring.	[232]
microRNAs	Behavior-related genes	Paternal stress led to alterations in sperm microRNA content and transgenerational effects on offspring behavior.	[233]
microRNAs	Metabolic genes	Sperm microRNAs were altered by diet and paternal obesity, influencing offspring metabolic health and susceptibility to metabolic disorders.	[234]
miRNAs	Fertility-related genes	Abnormal sperm miRNA profiles were associated with male infertility and could potentially be transmitted to offspring, impacting fertility and reproductive health.	[235]
piRNAs	Reproductive genes	Aberrant piRNA expression in sperm was linked to male infertility and may contribute to transgenerational effects on reproductive health and fertility.	[230]
